# The effectiveness of knowledge management systems in motivation and satisfaction in higher education Institutions: Data from Vietnam

**DOI:** 10.1016/j.dib.2023.109454

**Published:** 2023-07-28

**Authors:** Bui Thanh Khoa, Tran Trong Huynh

**Affiliations:** a Faculty of Commerce and Tourism, Industrial University of Ho Chi Minh City, Ho Chi Minh City, Viet Nam; b Mathematics Department, FPT University, Ha Noi, Viet Nam

**Keywords:** Knowledge acquisition, Knowledge utilization, Knowledge dissemination, Dataset, Vietnam

## Abstract

Knowledge management in higher education aims to increase the effectiveness of knowledge and intellectual capital by accomplishing three main goals: increasing task quality and efficiency, training human resources at all levels of operation, and expanding an organization's field knowledge base. This data's purpose was to shed light on how knowledge management influences the enthusiasm of university professors and their sense of job satisfaction. Knowledge acquisition, knowledge dissemination, and utilization are the three pillars of knowledge management systems that contribute to higher levels of academic staff's teaching motivation and satisfaction. A self-administered questionnaire collected this data from 676 academics in Vietnam. Knowledge management system improvements can be based on data analysis to improve faculty happiness and enthusiasm for academic staff in Higher Education Institutions.

**Specifications Table**

**Table:** 

Subject	Business, Management, and decision sciences
Specific subject area	Management Information Systems
Type of data	Tables
How the data were acquired	Survey data through the questionnaire, which has the research items based on prior studies. Adopted from Ngoc-Tan and Gregar (2018), the assessment of the knowledge management system included three constructs: knowledge acquisition (KNA), knowledge dissemination (KND), and knowledge utilization (KNU). Academic staff satisfaction (ASS), which included three items, was provided by Lee et al. (2000); and teaching motivation (four items, TEM) was based on research from Tang et al. (2016); Wilkesmann and Lauer (2020)
Data format	Raw
Description of data collection	A self-administered questionnaire was sent to 700 professors at higher education institutions in Vietnam. Data for this survey was gathered using “purposive sampling.” All participants have used the knowledge management system for at least three months, and their university has implemented a knowledge management system. After the first curation, 676 replies (96.57% of the total) were usable for further statistical examination.
Data source location	City/Town/Region: 5 most prominent cities in Vietnam (Ha Noi, Hai Phong, Da Nang, Ho Chi Minh City, Can Tho) Country: Vietnam
Data accessibility	Repository name: Mendeley Data Data identification number: 10.17632/tpgsnk4928.3 Direct URL to data: https://data.mendeley.com/datasets/tpgsnk4928

## 1 Value of the Data

• The data related to management information systems, especially in the field of knowledge management systems in education; therefore, this data will provide a better understanding of the influence of the knowledge management system on teaching motivation and teacher satisfaction.
• Through data, managers at higher education institutions can have policies in place to improve satisfaction and motivation for trainers.
• This data is only used to test hypotheses and evaluate relationships between research structures. However, this data can be used to assess the mediating role of teaching motivation or to examine the moderating role of demographic variables such as gender, age, expertise, and education level.

## 2 Objective

Improving the quality of education is an essential task for every country. Since the development of technology, improving the quality of higher education has been associated with the knowledge management system [1]. To understand the influence of knowledge management on the teaching motivation of lecturers, this study has collected data related to the perception of lecturers about the knowledge management system at higher education institutions, including knowledge acquisition, knowledge dissemination, and knowledge utilization. In addition, to better understand the teaching motivation of lecturers, the study also collected data on teaching satisfaction and motivation. From there, this study evaluated the relationship between these factors at higher education institutions in Vietnam.

## 3 Data Description

An online questionnaire was used to gather the data, which included a declaration on data ethics and confidentiality. The data has 31 variables except for the first column, the number of respondents. “applied” column describes respondents' knowledge management system application in their university. “timepoint” column is the last time using a knowledge management system. “KNA,” “KND,” “KNU,” “ASS,” and “TEM” columns point out the academic staff's agreement level regarding Knowledge Acquisition, Knowledge Dissemination, Knowledge Utilization, Academic Staff Satisfaction, and Teaching Motivation, respectively. Finally, participants’ gender, age, graduated major, and education level was collected in four variables, including “gender,” “age,” “major,” and “edule.”

Table 1 contains the categorical variables used for two screening and four demographic questions, whereas Tables 2 through 25 items use 5-point Likert scales [1: strongly disagree to 5: strongly agree] to record responses to the item statements. In this study, knowledge acquisition, knowledge dissemination, and knowledge utilization are the three dimensions of knowledge management systems that positively impact teaching motivation and academic staff satisfaction [2,3]; moreover, the more teachers’ motivation is, the more their satisfaction is in the high education institutions [4]. The measurement items and their respective codes are shown in Tables 1 and 2.

**Table 1:** Item coding for screening and demographic questions.

Question	Coding
**Is the knowledge management system applied in your university?** Yes No (stop survey)	1 2
**When did the last time you used a knowledge management system?** Less than one week One week ago One month ago Three months ago. Six months ago (stop survey) One year ago (stop survey) More than one year (survey stop)	1 2 3 4 5 6 7
**Gender** Male Female	1 2
**Age group** 24–30 31–35 36–40 40–45 > 45	1 2 3 4 5
**Major** Management Science Technical science Social science	1 2 3
**Education level** Bachelor Master Doctor/Ph.D.	1 2 3

**Table 2:** Item wordings for research constructs.

Code	**Knowledge Acquisition (KNA)**
KNA1	The free flow of information and ideas across different groups is actively encouraged and supported at my institution (faculties and administrative staff).
KNA2	My institution has a system set up to gather data from customers, employees, vendors, and competitors.
KNA3	My institution takes our feedback seriously and files it away for consideration.
KNA4	The policies at my institution strongly support staff members' pursuit of further education.
KNA5	My institution recognizes us for our innovative thinking and high level of skill.
KNA6	My institution has a network for receiving and sending data.
	**Knowledge Dissemination (KND)**
KND1	There are many places to study and share information at my institution.
KND2	The faculty and staff at my institution are familiar with the document.
KND3	My institution has a process in place for protecting original research.
KND4	Publications featuring the research conducted at my institution are available to the public.
KND5	My institution often hosts forums for academic discussion in the form of symposia, seminars, conferences, and workshops.
KND6	My institution stores its data in a variety of written formats, including bulletins and manuals.
KND7	My institution has centralized data storage areas that professors may immediately access.
	**Knowledge Utilization (KNU)**
KNU1	In order to create useful trends and insights for the future, my institution employs data analysis.
KNU2	Information is used to help my institution stay competitive and achieve vital industry standards.
KNU3	My institution takes the security of student data very seriously, both internally and externally.
KNU4	There are a variety of approaches used at my institution to broaden horizons and transfer learning to new contexts.
KNU5	My institution has an infrastructure in place for the screening, referencing, and integrating of information.
	**Academic Staff Satisfaction (ASS)**
ASS1	I have a strong commitment to the knowledge management initiatives at my workplace.
ASS2	Thanks to its dedication to knowledge management, I am glad they have a chance to further their education at this university.
ASS3	I am happy with how the institution handles knowledge management.
	**Teaching Motivation (TEM)**
TEM1	Knowledge gained via the institution's knowledge management procedures is essential for the education of its students.
TEM2	Thanks to the resources provided by the institution, I have become an expert in my field and can pass that knowledge on to my students.
TEM3	I hope that the issue will pique the curiosity of others.
TEM4	From my perspective, my teaching significantly impacts my students' eventual academic success.

Seven hundred professors at higher education institutions in Vietnam were sent the link to join the survey. As a result, there were 676 replies in the dataset (96.57% of the total). All respondents have used the knowledge management system in their university. Descriptive data for several demographic questions are included in Table 3, including frequency and percentage tests. In Table 4, the mean, standard deviation, kurtosis, and skewness tests for the multi-item scales were done for all items of research constructs. SPSS 28 was used to produce Tables 3 and 4.

**Table 3:** Frequencies statistics for screening and demographic questions.

Characteristic		Frequency	Percent
Knowledge management system last time using	Less than one week	146	21.6
	One week ago,	164	24.3
	One month ago,	187	27.7
	Three months ago,	179	26.5
Gender	Male	341	50.4
	Female	335	49.6
Age group	24–30	218	32.2
	31–35	202	29.9
	36–40	104	15.4
	41–45	62	9.2
	More than 45	90	13.3
Major	Management Science	232	34.3
	Technical science	226	33.4
	Social science	218	32.2
Education level	Bachelor	54	8.0
	Master	394	58.3
	Doctor/Ph.D.	228	33.7

**Table 4:** Descriptive statistics of the constructs’ items.

Item	Mean	Std. Deviation	Skewness		Kurtosis	
			Statistic	Std. Error	Statistic	Std. Error
KNA1	3.78	0.856	−0.632	0.094	0.446	0.188
KNA2	3.87	0.807	−0.496	0.094	0.103	0.188
KNA3	3.79	0.849	−0.402	0.094	0.119	0.188
KNA4	3.85	0.906	−0.521	0.094	0.035	0.188
KNA5	3.70	0.772	−0.571	0.094	0.896	0.188
KNA6	3.87	0.945	−0.900	0.094	0.859	0.188
KND1	3.94	0.955	−0.772	0.094	0.361	0.188
KND2	3.84	0.948	−0.742	0.094	0.351	0.188
KND3	3.95	0.937	−0.903	0.094	0.735	0.188
KND4	3.94	0.985	−0.868	0.094	0.616	0.188
KND5	3.88	1.017	−0.896	0.094	0.287	0.188
KND6	3.63	1.078	−0.549	0.094	−0.366	0.188
KND7	3.46	1.087	0.456	0.094	6.268	0.188
KNU1	3.65	0.867	−1.120	0.094	1.877	0.188
KNU2	3.68	0.834	−0.751	0.094	0.726	0.188
KNU3	3.83	0.952	−1.292	0.094	1.832	0.188
KNU4	3.77	0.925	−0.792	0.094	0.847	0.188
KNU5	3.55	0.855	−0.864	0.094	1.335	0.188
ASS1	4.05	0.990	−1.008	0.094	0.726	0.188
ASS2	3.97	1.100	−1.024	0.094	0.643	0.188
ASS3	3.96	1.014	−0.833	0.094	0.351	0.188
TEM1	3.86	1.060	−0.725	0.094	0.058	0.188
TEM2	3.85	1.052	−0.695	0.094	0.004	0.188
TEM3	3.94	0.999	−0.819	0.094	0.427	0.188
TEM4	3.80	1.046	−0.549	0.094	−0.146	0.188

The measurement model has tested reliability and validity. Table 5 points out that all scales have got reliability and convergent validity as Cronbach's Alpha (CA) and Composite Reliability (CR) are more significant than 0.7, Average Variance Extracted (AVE) is more extensive than 0.5, and the outer loading value is higher than 0.708. Moreover, The Heterotrait-Monotrait Ratio (HTMT) value proved that all constructs got the discriminant validity as HTMT is less than 0.85 [5].

**Table 5:** The reliability and validity of the measurement model.

Construct	CA	CR	AVE	Outer loading	HTMT			
					ASS	KNA	KND	KNU
ASS	0.857	0.913	0.778	0.801–0.922				
KNA	0.848	0.886	0.566	0.720–0.777	0.782			
KND	0.929	0.943	0.704	0.718–0.892	0.719	0.682		
KNU	0.913	0.935	0.741	0.844–0.885	0.651	0.607	0.625	
TEM	0.878	0.916	0.732	0.837–0.880	0.718	0.713	0.736	0.674

Furthermore, when using factor-based PLS-SEM algorithms, the variance inflation factors threshold (VIF) employed in tests should probably be a little higher than 3.3 to guarantee unbiased research instruments in the commonly used approach bias in the Partial Least Squares Structural Equation Model (PLS-SEM) [6]. As can be seen in Table 6, all the latent variables in this investigation had VIF values lower than 3.3, suggesting that the prevalent common method bias was not a problem.

**Table 6:** common method bias via VIF.

Relationship	VIF
ASS -> TEM	2.336
KNA -> ASS	1.773
KNA -> TEM	2.104
KND -> ASS	1.876
KND -> TEM	2.075
KNU -> ASS	1.646
KNU -> TEM	1.755

**Fig. 1:**
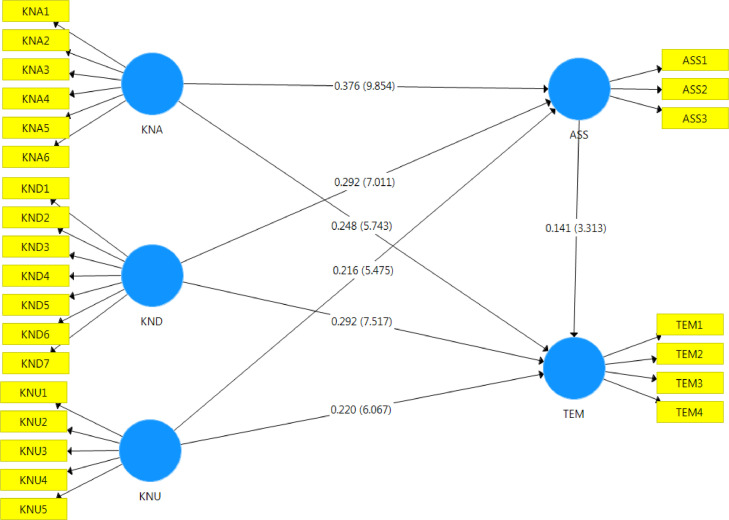
The PLS-SEM result.

Finally, the data can be used to establish the PLS-SEM, which pointed out that all research constructs have positive relationships as beta values are more significant than 0, and the p-value is less than 0.001. Fig. 1 shows the PLS-SEM result.

## 4 Experimental Design, Materials and Methods

The poll questions were revised from the prior study. Seven knowledge management professionals, senior professors, and researchers were surveyed for preliminary data to revise these items. The authors instructed the focus group using a discussion guideline sent to the participants. Expert consensus supports using the research model's components, and the research questions used to test those constructs were adopted to the study's objective.

The questionnaire's scales were all taken directly from published research. Three constructs, Knowledge Acquisition (KNA) with six items, Knowledge Dissemination (KND) with seven items, and Knowledge Utilization (KNU) with five items, were adopted to evaluate the knowledge management system [7,8]. The Education Criteria of the Malcolm Baldrige National Quality Award, taken from Lee, Lo, Leung and Ko [9], serve as the basis for the three-item measured Academic Staff Satisfaction (ASS) scale; and teaching motivation (4 items, TEM) as an example of extrinsic motivation adopted from Wilkesmann and Lauer [10], Tang, Wong and Cheng [11].

Seven hundred academic staffs lecturing in Vietnam's universities from the five most prominent cities in Vietnam (Ha Noi, Hai Phong, Da Nang, Ho Chi Minh City, Can Tho) were given a questionnaire they may fill out in their own time. Based on the propose of Brick and Tourangeau [12], this study used two methods to reduce the non-response bias. Firstly, the combination of modes of data collection, including online and offline surveys, was used. The paper questionnaire was distributed directly to the universities via mail for the lecturers, and the online questionnaire was sent via the lecturer's email. Secondly, a monetary incentive through the electronic wallet was adopted to encourage the lecturer's response after they finished the questionnaire. That is why the high response rate (more than 96%).

The research team used "purposive sampling" to collect the data they needed for the study. Three months of teaching experience with a knowledge management system are prerequisites for participation. Questions such as, "Is the knowledge management system applied in your university?" (Q1) and “When did the last time you used a knowledge management system?” (Q2). If Q1 is answered with "No," the poll will end; if Q2 is answered with a number less than three months, the survey will also end.

This data analysis was based on the PLS-SEM; therefore, researchers using PLS-SEM should do power studies or depend on heuristics like the inverse square root technique [13,14]. With a significant level of 5% and a minimum path coefficient of 0.141, as shown in Fig. 1, the minimum sample size is given by $n_{min}>{\left(\frac{2.486}{|p_{min}|}\right)}^{2}$; therefore, *n_min_* > 310.86. After the first round of curation, 676 responses (or 96.57% of the total) were of sufficient quality for statistical analysis.

After the data was gathered, it was reviewed for accuracy. All scales met the reliability and validity standards outlined in the literature. SPSS 28 and SmartPLS 3.8 were used to thoroughly examine the data and ensure that the AVE and the CR were within the ranges recommended by the literature. Next, the HTMT technique was used to test the discriminant validity as well as VIF analysis to test the common method bias. Finally, PLS-SEM was developed to check the hypotheses and relationships between the research constructs.

## Ethics Statements

Each author has followed the guidelines established by the Industrial University of Ho Chi Minh City's ethical committee. Before participating in the research, all participants were fully briefed on its nature and purpose. All participants gave their informed consent and can no longer be contacted. No Institutional Review Board (IRB) permission was necessary.

## CRediT authorship contribution statement

**Bui Thanh Khoa:** Conceptualization, Writing – original draft, Visualization, Investigation, Supervision, Software, Validation. **Tran Trong Huynh:** Methodology, Data curation, Writing – review & editing.

## Data Availability

The effectiveness of knowledge management systems in motivation and satisfaction in higher education Institutions (Original data) (Mendeley Data).

## Appxendix Supplementary materials

Image, application 1

Image, application 2

Image, application 3
