# Supplementary material for: The effectiveness of knowledge management systems in motivation and satisfaction in higher education Institutions: Data from Vietnam
Source: Data Brief. 2023 Jul 28;49:109454. doi: 10.1016/j.dib.2023.109454 (PMC10415701; doi:10.1016/j.dib.2023.109454)
Supplement: Supplementary file 2 [file mmc2.pdf]

NQUESTIONNAIRE

Dear Professors!

My name is Bui Thanh Khoa; I am a researcher at the Industrial University of Ho Chi Minh city

I am working on the paper entitled “The effectiveness of knowledge management systems in motivation and satisfaction in higher education Institutions: Data from Vietnam”. The success of the project depends on your answers to this survey. The information you provide will be confidential and used only for academic research.

If you have any questions, please get in touch with us at email buithanhkhoa@iuh.edu.vn

Thank you very much for your interest and time in participating in this survey.

I. Screening questions

1. Is the knowledge management system applied in your university?

☐ Yes

☐ No (stop survey)

2. When did the last time you use a knowledge management system?

☐ Less than one week

☐ One week ago

☐ One month ago

☐ Three months ago.

☐ Six months ago (stop survey)

☐ One year ago (stop survey)

☐ More than one year (survey stop)

II. Main questions

Please indicate your opinion by circling (or marking an X) the number that best describes your level of agreement with each of the following statements: 1 = Totally disagree; 2 = Disagree; 3 = Neutral; 4 = Agree; 5 = Totally agree.

Knowledge Acquisition (KNA)					
The free flow of information and ideas across different groups is actively encouraged and supported at my institution (faculties and administrative staff).	1	2	3	4	5

My institution has a system set up to gather data from customers, employees, vendors, and competitors.	1	2	3	4	5
My institution takes our feedback seriously and files it away for consideration.	1	2	3	4	5
The policies at my institution strongly support staff members' pursuit of further education.	1	2	3	4	5
My institution recognizes us for our innovative thinking and high level of skill.	1	2	3	4	5
My institution has a network for receiving and sending data.	1	2	3	4	5
Knowledge Dissemination (KND)					
There are many places to study and share information at my institution.	1	2	3	4	5
The faculty and staff at my institution are familiar with the document.	1	2	3	4	5
My institution has a process in place for protecting original research.	1	2	3	4	5
Publications featuring the research conducted at my institution are available to the public.	1	2	3	4	5
My institution often hosts forums for academic discussion in the form of symposia, seminars, conferences, and workshops.	1	2	3	4	5
My institution stores its data in a variety of written formats, including bulletins and manuals.	1	2	3	4	5
My institution has centralized data storage areas that professors may immediately access.	1	2	3	4	5
Knowledge Utilization (KNU)					
In order to create useful trends and insights for the future, my institution employs data analysis.	1	2	3	4	5
Information is used to help my institution stay competitive and achieve vital industry standards.	1	2	3	4	5
My institution takes the security of student data very seriously, both internally and externally.	1	2	3	4	5
There are a variety of approaches used at my institution to broaden horizons and transfer learning to new contexts.	1	2	3	4	5
My institution has an infrastructure in place for the screening, referencing, and integrating of information.	1	2	3	4	5
Academic Staff Satisfaction (ASS)					
I have a strong commitment to the knowledge management initiatives at my workplace.	1	2	3	4	5
Thanks to its dedication to knowledge management, I am glad they have a chance to further their education at this university.	1	2	3	4	5
I am happy with how the institution handles knowledge management.	1	2	3	4	5
Teaching Motivation (TEM)					
Knowledge gained via the institution's knowledge management procedures is essential for the education of its students.	1	2	3	4	5
Thanks to the resources provided by the institution, I have become an expert in my field and can pass that knowledge on to my students.	1	2	3	4	5
I hope that the issue will pique the curiosity of others	1	2	3	4	5
From my perspective, my teaching significantly impacts my students' eventual academic success.	1	2	3	4	5

III. Demographic questions

Please indicate your opinion by circling (or marking an X) the number that best describes your demographic

Gender	Male	1
	Female	2
Age group	24 - 30	1
	31 - 35	2
	36 - 40	3
	41 - 45	4
	> 45	5
Major	Management Science	1
	Technical science	2
	Social science	3
Education level	Bachelor	1
	Master	2
	Doctor/Ph.D.	3
