# Supplementary material for: The effectiveness of knowledge management systems in motivation and satisfaction in higher education Institutions: Data from Vietnam
Source: Data Brief. 2023 Jul 28;49:109454. doi: 10.1016/j.dib.2023.109454 (PMC10415701; doi:10.1016/j.dib.2023.109454)
Supplement: Supplementary file 3 [file mmc3.docx]

**CODEBOOK**

| **Code** | **Question** | **Coding** |
| --- | --- | --- |
| applied | **Is the knowledge management system applied in your university?** |  |
|  | Yes | 1 |
|  | No (stop survey) | 2 |
| timepoint | **When did the last time you used a knowledge management system?** |  |
|  | Less than one week | 1 |
|  | One week ago | 2 |
|  | One month ago | 3 |
|  | Three months ago. | 4 |
|  | Six months ago (stop survey) | 5 |
|  | One year ago (stop survey) | 6 |
|  | More than one year (survey stop) | 7 |
| gender | **Gender** |  |
|  | Male | 1 |
|  | Female | 2 |
| age | **Age group** |  |
|  | 24 - 30 | 1 |
|  | 31 - 35 | 2 |
|  | 36 - 40 | 3 |
|  | 40 - 45 | 4 |
|  | > 45 | 5 |
| major | **Major** |  |
|  | Management Science | 1 |
|  | Technical science | 2 |
|  | Social science | 3 |
| edule | **Education level** |  |
|  | Bachelor | 1 |
|  | Master | 2 |
|  | Doctor/Ph.D. | 3 |
|  | **Knowledge Acquisition (KNA)** | |
| KNA1 | The free flow of information and ideas across different groups is actively encouraged and supported at my institution (faculties and administrative staff). | 1. Strongly disagree 2. Disagree 3. Neutral 4. Agree 5. Total agree |
| KNA2 | My institution has a system set up to gather data from customers, employees, vendors, and competitors. |  |
| KNA3 | My institution takes our feedback seriously and files it away for consideration. |  |
| KNA4 | The policies at my institution strongly support staff members' pursuit of further education. |  |
| KNA5 | My institution recognizes us for our innovative thinking and high level of skill. |  |
| KNA6 | My institution has a network for receiving and sending data. |  |
|  | **Knowledge Dissemination (KND)** | |
| KND1 | There are many places to study and share information at my institution. | 1. Strongly disagree 2. Disagree 3. Neutral 4. Agree 5. Total agree |
| KND2 | The faculty and staff at my institution are familiar with the document. |  |
| KND3 | My institution has a process in place for protecting original research. |  |
| KND4 | Publications featuring the research conducted at my institution are available to the public. |  |
| KND5 | My institution often hosts forums for academic discussion in the form of symposia, seminars, conferences, and workshops. |  |
| KND6 | My institution stores its data in a variety of written formats, including bulletins and manuals. |  |
| KND7 | My institution has centralized data storage areas that professors may immediately access. |  |
|  | **Knowledge Utilization (KNU)** | |
| KNU1 | In order to create useful trends and insights for the future, my institution employs data analysis. | 1. Strongly disagree 2. Disagree 3. Neutral 4. Agree 5. Total agree |
| KNU2 | Information is used to help my institution stay competitive and achieve vital industry standards. |  |
| KNU3 | My institution takes the security of student data very seriously, both internally and externally. |  |
| KNU4 | There are a variety of approaches used at my institution to broaden horizons and transfer learning to new contexts. |  |
| KNU5 | My institution has an infrastructure in place for the screening, referencing, and integrating of information. |  |
|  | **Academic Staff Satisfaction (ASS)** | |
| ASS1 | I have a strong commitment to the knowledge management initiatives at my workplace. | 1. Strongly disagree 2. Disagree 3. Neutral 4. Agree 5. Total agree |
| ASS2 | Thanks to its dedication to knowledge management, I am glad they have a chance to further their education at this university. |  |
| ASS3 | I am happy with how the institution handles knowledge management. |  |
|  | **Teaching Motivation (TEM)** | |
| TEM1 | Knowledge gained via the institution's knowledge management procedures is essential for the education of its students. | 1. Strongly disagree 2. Disagree 3. Neutral 4. Agree 5. Total agree |
| TEM2 | Thanks to the resources provided by the institution, I have become an expert in my field and can pass that knowledge on to my students. |  |
| TEM3 | I hope that the issue will pique the curiosity of others. |  |
| TEM4 | From my perspective, my teaching significantly impacts my students' eventual academic success. |  |
